# The Institutional Library

**Published:** 1916-06-03

**Authors:** 


### THE INSTITUTIONAL LIBRARY.

**School Sanatoria and Infirmaries.** By Keith D. Young, F.R.I.B.A. (Adlard and Son and West Newman, Bartholomew Close, London, E.C.)

A paper read last November before the Medical Officers of Schools' Association by Mr. Keith D. Young, F.R.I.B.A., and published in the February number of *School Hygiene*, has been issued as a pamphlet. Mr. Young puts forward plans both for a sanatorium and an infirmary for a supposed school of 300 boarders. As he says, "It is not possible to devise a plan so elastic that it could be readily altered to suit all conditions." It is not pretended that simply by adding or taking away a floor or any other unit the plans can be adapted to the requirements of every school. The aim of the pamphlet is to provide a guide for those who have to design or criticise plans for the sanatorium or infirmary of any actual school. The most inexperienced would not fail to consider, and probably estimate justly, the number of cases that the building should accommodate; it is not likely that due provision would not be made for kitchen and other offices and for the permanent staff. Here advice is readily obtained and assimilated. But there are more intricate problems than these, and details of arrangement that may easily be overlooked till a test reveals too late the deficiency. The number of nurses, for instance, is a variable quantity. The plans do not provide bedrooms for all the nurses that would be required if the sanatorium were full, but this point has not been neglected. Accommodation could probably be found outside for occasional nurses; but, if they have to leave the building, rooms must be provided for disinfection and for changing clothes. Again, it is not enough merely to provide wards containing the requisite number of beds. The problem is by no means so simple. The simultaneous occurrence of different kinds of infectious complaints must be considered; it must be possible to keep them entirely separate; not only separate wards must be provided, but separate access to these wards—separate staircases, lifts, and day-rooms, separate bathrooms and dressing-rooms for discharging patients. All these points are raised in the pamphlet; anyone who reads it through with actual plans before him could hardly fail to note if those plans were anywhere deficient.

Practical experience shows that it is enough to provide for two different epidemics at once, and Mr. Young has perhaps been too lavish in his provision of single-bed wards; but it certainly is an advantage to have all the patients on the same floor if numbers permit, and if all the floors are to be alike the single-bed wards must be repeated. An interesting paragraph at the end of the pamphlet discusses the question of cubic space per bed. Official formulæ need sometimes to be revised in the light of further knowledge. At a time when methods of ventilation were other than they are now the official allowance per bed was 2,000 cubic feet, and floor-area has long been fixed at 144 square feet. But it is now held that all height above 10 feet is negligible for purposes of ventilation. The requirements of cubic space and floor-area, therefore, no longer agree, and Mr. Young considers that both might safely be reduced below the regulation allowance. Windows, floor and wall surfaces, and heating are all touched upon. Anyone who has to consider the erection of a new school sanatorium or infirmary, or the enlargement or alteration of an old one, will find it well worth while to read these ten pages of condensed and suggestive matter on an interesting subject.

**The Pathology of Tumours.** By E. H. Kettle, M.D. (London: H. K. Lewis and Co. 1916. Pp. 224. Illust., 126. Price 10s. 6d. net.)

This is a book which should become very popular among medical students and young qualified men preparing for higher examinations. It provides them with something which for many years they have not had—a sound, up-to-date, not too advanced review of modern research and modern theories upon the intensely interesting and difficult subject of tumour pathology. It fills, in fact, the place which sixteen or eighteen years ago the text-book of Sutton and Giles occupied. The treatment is elementary, and both lucid and free from discursiveness. Dr. Kettle is at his best in pointing out the inconsistencies and limitations of many hypotheses which are confidently upheld by some authorities, especially their own begetters. His criticisms are mainly destructive, that is, rather than constructive; but this is a positive advantage because so much nonsense has been talked and written on the subject of tumours, both by clinicians and by pathologists, and because in our present state of knowledge negative conclusions have been much more abundantly supplied by the research of the last few years than have positive ones. Clinical topics are rightly kept well in the background, though they are not excluded altogether; and histological methods are omitted entirely, on the ground that there is nothing peculiar to the histology of tumours. The classification adopted is tentative and not too cumbrous; and the illustrations are really illustrative. In spite of the unpropitious times, we are confident that this book will find a wide public among practitioners and students; we may add that it is too technical to cater for the layman.

**Incompatibility in Prescriptions and How to Avoid It.** By Thos. Stephenson, Ph.C., F.C.S. (Edinburgh: *The Prescriber* Offices. 1915. Pp. 32. Price 1s. net.)

Whether for good or ill, the number of doctors who write prescriptions instead of themselves dispensing medicines for their patients has been very greatly increased by the National Insurance Acts. For their own credit, as well as in the interests of the patients, it is desirable that these prescriptions should not contain errors which show ignorance of the physical and chemical properties of the substances they contain. Apart from other unhappy consequences, such errors are apt to provoke what an old writer calls the "sneering of the apothecary's boy." Mr. Stephenson's pamphlet may thus be said to have had a special audience prepared for it by the operation of the Insurance Acts. It is a well-ordered presentation of the errors in combination into which prescribers are likely to slip. Most of us were warned against these mistakes in our student days, but memories become dulled by time and by absence of opportunity for realising in practice the physical and chemical characters of the remedies we prescribe. This last-mentioned result is one of the unfortunate consequences of a non-dispensing medical practice, though this is not to say that there is nothing to be urged on the other side. Taking the state of matters as they stand to-day, there is certainly an opportunity for reminding medical practitioners that certain combinations of remedies are inelegant or even dangerous, and Mr. Stephenson's pages utilise this opportunity with considerable success.

**The History of the Fabian Society.** By Edward R. Pease. With Portraits, Appendices, and Index. (London : A. C. Fifield. Price 5s. net.)

There are two good reasons why hospital men should read this entertaining volume. First, it deals with the origin and varied activities of a society of Socialists who have led the van in putting the case for a municipal hospital system, and it is always well to study one's opponent's case. Secondly, institutional workers are the sole class in the community who have ever visited Utopia, the stony paths to which the Fabians have attempted to point out. For the well-managed institution, especially the well-managed hospital, is the typical example of Society's energies on the utopian or constructive side. With the number of visitors who come to them, hospital men become prone to take as a matter of course the expressions of surprise and delight which are produced in a stranger by a first view of the cleanliness, order, and arrangement prevailing in hospitals. No intelligent person can ever forget the impressions of such a first visit. Institutional progress is the realisation of Utopias on a small scale, for this or that purpose. It is this fact which gives its interest to the present volume; for, apart from the glimpses here provided of the early work of the group of since famous men who formed the society's first leaders, this history of the Fabian Society is the history of ideas, of theories, many of which, in their practical applications, are seen in the bricks and mortar of institutional life. If it does not interest him, the reader can ignore the Fabian economics. We can ignore the Socialism, the chief intellectual exposition of which in England it has been the Fabian's business to provide; but it will do him good to read how the tracts on school clinics, on infantile mortality, on the milk supply, on the treatment of the sick under the Poor Law were compiled. The "case against the C.O.S." should certainly not be missed for its humour and its trenchancy. The Fabian case and presentment of facts has always had an outstanding merit. It was never dull. It generally knew the facts; and if it made a mistake it made it thoroughly. Mr. Pease's history has all these virtues. It is full of humour, of facts, and of amusing footnotes. The illustrations are distinctly curious. In binding and printing the publisher has treated his readers well.

**Home Nursing.** By Edith Newsome, M.R.B.N.A. (London : The Scientific Press, Ltd. Price 2s. 6d. net.)

This book, while too ambitious in its range for use as a text-book by the neophyte of Red Cross nursing, is one that might well be added to the library of all those interested in the art and practice of nursing. The arrangement of the book, the paper, printing, and illustrations are all good. The book is full of information, but contains jerky sentences and exhibits a sparing use of words. We miss the usual friendly advice of the duty of the nurse to the doctor, and we feel it unnecessary for a nurse to express an opinion on the various points in relation to the abnormal pulse, and some other matters which are hardly within her orbit. The diet of the invalid should be in the hands of the medical attendant, the nurse taking care that she receives explicit directions as to the quality and quantity of nourishment the patient should be given, and when nursing a case of enteric fever this is of vital importance. There are some points that require revision in another edition. The remarks on p. 107 under the heading "Tea" savour of nonsense. It is an error to peptonise milk by allowing peptonisation to go on for one and a half or two hours; unpleasant, bitter products will be formed. In the instruction given for bandaging the breast, the error (in our opinion) of taking the bandage over the shoulder of the same, instead of the opposite, side to the breast being bandaged, is perpetrated in this book, as it is in some other nursing manuals. There is no index. In the piping times of peace, with leisure at her disposal, Nurse Newsome will doubtless revise and rewrite her book, substituting for the existing staccato phraseology of *le livre de cuisine* one more worthy of her subject.

**Sight-Testing Made Easy.** By W. Wright Hardwicke, M.D. St. And., M.R.C.P., L.R.C.S. Ed. (London: J. and A. Churchill. 1910. Pp. 80. 2s. 6d. net.)

Dr. Hardwicke's manual, having been revised and in some parts rewritten, has now reached its third edition. It is doubtless by this time well known to many as a reliable and readable introduction to the study of the refraction of the eye. Part I., in which mainly the optics of the subject are dealt with in an elementary manner, and which occupies half the subject-matter of the book, is particularly straightforward and clear; as regards the remaining two parts we think it a pity that greater prominence has not been given and more space devoted to Part III. (Objective Sight-testing by Retinoscopy) at the expense of Part II. (Subjective Sight-testing). The practitioner who makes a practice of prescribing glasses, and who has taken the trouble to master the art of retinoscopy, will not only obtain accurate results in all cases, as opposed to mere approximate makeshifts in most cases of astigmatism, but will also save an immense amount of time and avoid much tedious and unprofitable labour. We do not, however, agree with Dr. Hardwicke's statement that retinoscopy can be easily learnt by practice on the model eye; in our opinion practice on the human eye is essential. As an introduction to larger works on the subject we can, as heretofore, confidently recommend the little volume.

**My Diary in Serbia, from April to November, 1915.** By Monica M. Stanley (attached to the Stobart Field Hospital in Serbia). (London: Simpkin, Marshall, Hamilton, Kent and Co. Price 2s. net.)

A record of incidents is not a diary, for incidents happen to us all, but the gift of the inspired gossip which makes a good diarist is given to very few. Yet, if we compare a diary of hospital experience, like Walt Whitman's "Specimen Days in America," with so different a book as that of Pepys, we shall see that inspired gossip is the basis of both. Though the author of this little book is not inspired, and a tyro at writing, her pages have patches of present interest from such glimpses as she gives of Mrs. Dearmer's last illness, or, in another vein, of Sir T. Lipton's visit with an escort of reporters; or, again, of a Serbian engagement ceremony. Occasionally a personal note is struck. But these personal touches are too few to make a really good diary.

**The Medical Who's Who, 1916.** (London : Fulton-Manders Publishing Company. Pp. 1,140. 10s. 6d. net.)

This work aims at being a list of the more notable physicians and surgeons of Great Britain and Ireland, but on looking through the pages one fails to discover the names of many well-known medical men, and notices that the length of the paragraph devoted to each name is no guide to the eminence of the person described. Though the last-mentioned phenomenon is common to most "Who's Whos," the total omission of leading names detracts from the value of the book as a work of reference, but when this obvious drawback has been put right the publication, which contains information not readily obtainable elsewhere, will be greatly improved.

